# Effects of spatial fragmentation on the elevational distribution of bird diversity in a mountain adjacent to urban areas

**DOI:** 10.1002/ece3.9051

**Published:** 2022-07-04

**Authors:** Wei Liu, Dandan Yu, Sijia Yuan, Jianfeng Yi, Yun Cao, Xiufen Li, Haigen Xu

**Affiliations:** ^1^ Nanjing Institute of Environmental Science Ministry of Ecological Environment Nanjing China; ^2^ College of Marine Life Sciences and Frontiers Science Center for Deep Multispheres and Earth System Ocean University of China Qingdao China; ^3^ Jiangsu Key Laboratory for Biodiversity and Biotechnology College of Life Sciences Nanjing Normal University Nanjing China; ^4^ Taian Taishan Mountain Beauty Spot Management Committee Taian China

**Keywords:** bird diversity, elevational distribution, montane, urbanization

## Abstract

The biodiversity in mountainous ecosystems is high but is threatened by rapid environmental change. Urbanization and other anthropogenic factors in the mountains can affect land use and spatial fragmentation. Moreover, patterns of habitat are closely related to elevation and have a major effect on montane biodiversity. The aim of this study was to analyze the effects of spatial fragmentation on the vertical distribution pattern of bird diversity by characterizing the structure of the bird community, species diversity, and landscape factors at different altitudes. From 2016 to 2019, this study made a four years of continuous monitoring of the breeding birds. The result indicated that Mount Tai harbored a high bird diversity. Bird richness, abundance, and Shannon‐Wiener index decreased with latitude in Mount Tai monotonically. Moreover, the structure of bird communities varied along altitudinal gradients, and some special species were supported in different elevational bands due to the environmental filtering. Road density, number of habitat patches, patch density, and the percentage of forest were significantly related to bird diversity. Sufficient habitat and more patches in the low‐mountain belt supported higher bird diversity. The middle‐mountain belt and high‐mountain belt showed contrasting patterns. Our results highlight the effects of on‐going urbanization and human activities on montane biodiversity and emphasize the need for artificial habitats in the mountains to be managed.

## Graphical abstract

This study analyzed the 4 years continuous field bird survery to unravel the effects of spatial fragmentation on the elevational distribution of bird diversity. Human disturbance and landscape diversity play a vital role in the pattern of bird diversity in Mount Tai, which emphasize the need for the management of artifical habitats .

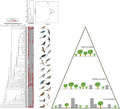

## 1 INTRODUCTION

Mountainous ecosystems are some of the most important and vulnerable ecosystems worldwide because of their rich biodiversity (McCain, 2009; Quintero & Jetz, 2018). Mountain ecosystems support more than 85% of all terrestrial biodiversity including plants, animals, and macromycetes, and approximately half of the global biodiversity hotspots with 13%–25% of all inland area (Peters et al., 2019; Quintero & Jetz, 2018; Spehn et al., 2011; Steinbauer et al., 2018). Over evolutionary time, mountain systems are considered as many of the world's centers and potential refugia of endangered species today, playing a vital role in maintain the high global or regional biodiversity (Antonelli et al., 2018; Hu et al., 2021; Rahbek, Borregaard, Antonelli, et al., 2019a). For example, the European Alps and Qinling Mountains are generally considered to harbor many threatened and endangered animals (Hu et al., 2021; Rahbek, Borregaard, Colwell, et al., 2019b). However, with the rapid development of urbanization and increasing land‐use intensity, loss of biodiversity continues at unprecedented rates in human history, especially in tropical mountains (Butchart et al., 2010; Colwell et al., 2004; Peters et al., 2019; Xu et al., 2021). Our understanding of the spatiotemporal maintenance of biodiversity in mountain remains poor, relatively little is known about how land‐use change and human activities influence biodiversity along elevational gradients. Thus, this study was designed to figure out the mechanism that how the habitat fragmentation affect biodiversity along elevational gradients in the context of urbanization. With the decline in terrestrial biodiversity, understanding population structure and spatiotemporal distribution patterns in mountainous ecosystems is important for formulating conservation strategies (Barbier et al., 2018).

There are many valuable studies illustrating the impacts or mechanism of different environmental factors on biodiversity, and the elevational change of biodiversity has always been a research hotspot over recent decades (Colwell et al., 2004; Rahbek, Borregaard, Colwell, et al., 2019b). In mountainous areas, there is a vertical gradient in species composition because of variation in abiotic conditions. Mountain regions with a high level of geological heterogeneity could support higher levels of species spatial turnover and endemic forms, particularly among plants (Rahbek, Borregaard, Antonelli, et al., 2019a). Multiple environmental factors, such as climate, hydrology, slope, habitat type, man‐made interference, and landform, affect distribution patterns of biodiversity in mountainous ecosystems (Jetz et al., 2012; Mccain, 2005). The elevational distribution patterns of insects, moss, bats, birds, vascular plants, and other taxa often show unimodal or monotonic change due to environmental factors (Mccain, 2007b; Song et al., 2015; Tabarelli et al., 1999). Mountain ecology has a potential importance on elevational biodiversity. For example, the topography, climate change, and mineralogical composition in mountain affect the survival of many species, plant physiology, and primary productivity (Emel et al., 2021; Steinbauer et al., 2018; Peters et al., 2019; Rahbek, Borregaard, Antonelli, et al., 2019a; Woodbridge et al., 2021).

Nowadays, the high spatial heterogeneity of ecological and environmental variables characteristic of mountains are suffering the inference of human activities and land use change (Pan et al., 2019; Peters et al., 2019). The level of species diversity and distribution pattern is associated with landscape fragmentation and habitat loss (Emel et al., 2021; Woodbridge et al., 2021). The effect on biodiversity has been exacerbated by the intensification of anthrogenic activities such as deforestation and urbanization, and these have led to significant land‐cover changes (García‐Llamas et al., 2018; Katayama et al., 2014; Pan et al., 2019). Recent studies and syntheses have increasingly implicated that human activities are strongly related to the changes in biodiversity, at both genetic and species levels (Emel et al., 2021; Woodbridge et al., 2021). Previous study in Andean mountain also demonstrated that human disturbance has been much less extreme in the lowlands, and the diversity of lowland habitats is much higher than it is anywhere along adjacent elevational transects (Butchart et al., 2010; Colwell et al., 2004; Terborgh et al., 1990).

Habitat features, especially that resulting from habitat fragmentation and changes in land utilization, playing an important role in population stability and biodiversity (Fahrig, 2017; Fahrig et al., 2019; Fletcher et al., 2018). Natural habitats are continuously being degraded and lost because of anthropogenic activities, and the ecological mechanisms have become a major focus of research (Butchart et al., 2010; Pereira et al., 2010; Pimm et al., 2014). Tourism, plantation, land development, and species invasion all affect natural habitats and lead to variation in food production, intensity of disturbance, vegetation, and landform (Lele et al., 2020; Peh et al., 2005; Xu et al., 2018). For montane birds, habitat fragmentation and heterogeneity can restrict the ranges of activity, affect levels of biodiversity, and make birds more vulnerable to environmental change (Lele et al., 2020). Continuous observations of birds in New Jiangwan Town in Shanghai have revealed that the mean species abundance (MSA) is strongly negatively correlated with the degree of urbanization and loss of natural habitats (Xu et al., 2018). Multiple hypotheses are developed to explain the spatial–temporal pattern of bird diversity in mountain system, such as mid‐domain effect hypothesis, climate hypothesis, space hypothesis, species–area relationship hypothesis, and habitat amount hypothesis (Colwell et al., 2004; Mittelbach et al., 2010; Rybicki et al., 2020). Human disturbance and habitat fragmentation were rarely considered by these hypotheses. There is thus a need to analyze the effects of human activities and urbanization on bird diversity in mountainous ecosystems (Rybicki et al., 2020).

As montane birds are highly sensitive to habitat changes (Soh et al., 2006), many studies have explored the structure of bird communities and the distribution patterns of montane birds under different degrees of disturbance (Fletcher et al., 2018; Harris & Pimm, 2010; Wu et al., 2010). Habitat environment often shows obvious changes over short distances in mountainous ecosystems, and birds have developed specialized adaptations (Quintero & Jetz, 2018). Rapid spatial changes in landform, vegetation, and disturbance could lead to the changes in the structure, richness, and diversity of bird communities in mountainous areas. Land use and altitude can restrict the distribution of biota and hinder bird activity (Harris & Pimm, 2010; Jetz et al., 2007). The environment at different altitudes strongly affects the ecological, evolutionary, physiological, and protective function of biodiversity (Pounds et al., 1999; Pounds et al., 2006).Functionally similar and closely related montane birds are clustered into groups under the constraints of regional habitats and environmental factors (Fahrig, 2003; Gao et al., 2018; Haddad et al., 2015; Pounds et al., 2006). Some birds tend to live in the high elevation, such as White‐bellied Redstart (*Luscinia phaenicuroides*) in the forest in Mount Tai.

Here, the elevational distribution pattern of birds in Mount Tai is analyzed by integrating data on landscape factors with data on montane biodiversity, and we expect to study the joint effects of human disturbance and habitat fragmentation on biological communities and biodiversity patterns. Elevation gradients in mountain regions are invaluable as a natural laboratory for the empirical testing of the hypothesized framework for biodiversity patterns and their links to human disturbance. This study can be divided into three parts: (1) Based on the continuous field survey, bird data were collected to analyze the community structure, diversity, and distribution pattern of birds. Species, individuals, and disturbance are essential to monitor the variation of bird diversity; (2) To ensure the accuracy and reliability of land‐use data, supervision classification was utilized to interpret the remote sensing. The acquisition of landscape matrix was also analyzed by software Fragstats V4.3. What is more, environmental factors, including roads and elevation, were obtained to generate multiscale data; (3) Combined the landscape features and bird data, the relationship between them was characterized at different altitudes. It is necessary to figure out the bird diversity patterns along elevational gradients and the influence of environmental variables on bird diversity.

## 2 METHODS

### 2.1 Study area

Mount Tai is located in the middle of Shandong Province (36°05′ ~ 36°75′N, 116°50′ ~ 117°24′E) on the edge of North China, a priority region for biodiversity protection. Mount Tai, a UNESCO World Heritage Site, has profound ecological and cultural value. The terrain of Mount Tai is precipitous and open, which has varied climate and topography. The elevation changes sharply, and the height of its peak, the Heavenly Emperor, is 1545 m above sea level. As a UNESCO World Heritage Site, Mount Tai features rich ecological and cultural resources. With the development of urbanization, Mount Tai might suffer from varying degrees of human disturbance and land development at different altitudes. Thus, Mount Tai is a suitable region for studying the effect of spatial fragmentation on biodiversity with urbanization.

There are obvious distinctions among the different vertical belts with different climate and vegetation. The habitat types from low to high elevation include deciduous forest, broad‐leaf coniferous mingled forest, coniferous forest, and high‐mountain scrub‐grassland (Wang & Li, 2013). Based on altitude and vegetation, Mount Tai can be separated into three vertical belts: the low‐mountain belt (altitude lower than 500 m), middle‐mountain belt (altitude from 500 to 1200 m), and high‐mountain belt (altitude above 1200 m) (Du, 1985). From 1986 to 2001, the biodiversity and natural habitat have declined and tourism (and the degree of human interference generally) has increased (Xiao & Luo, 2005).

### 2.2 Field survey

**FIGURE 1:**
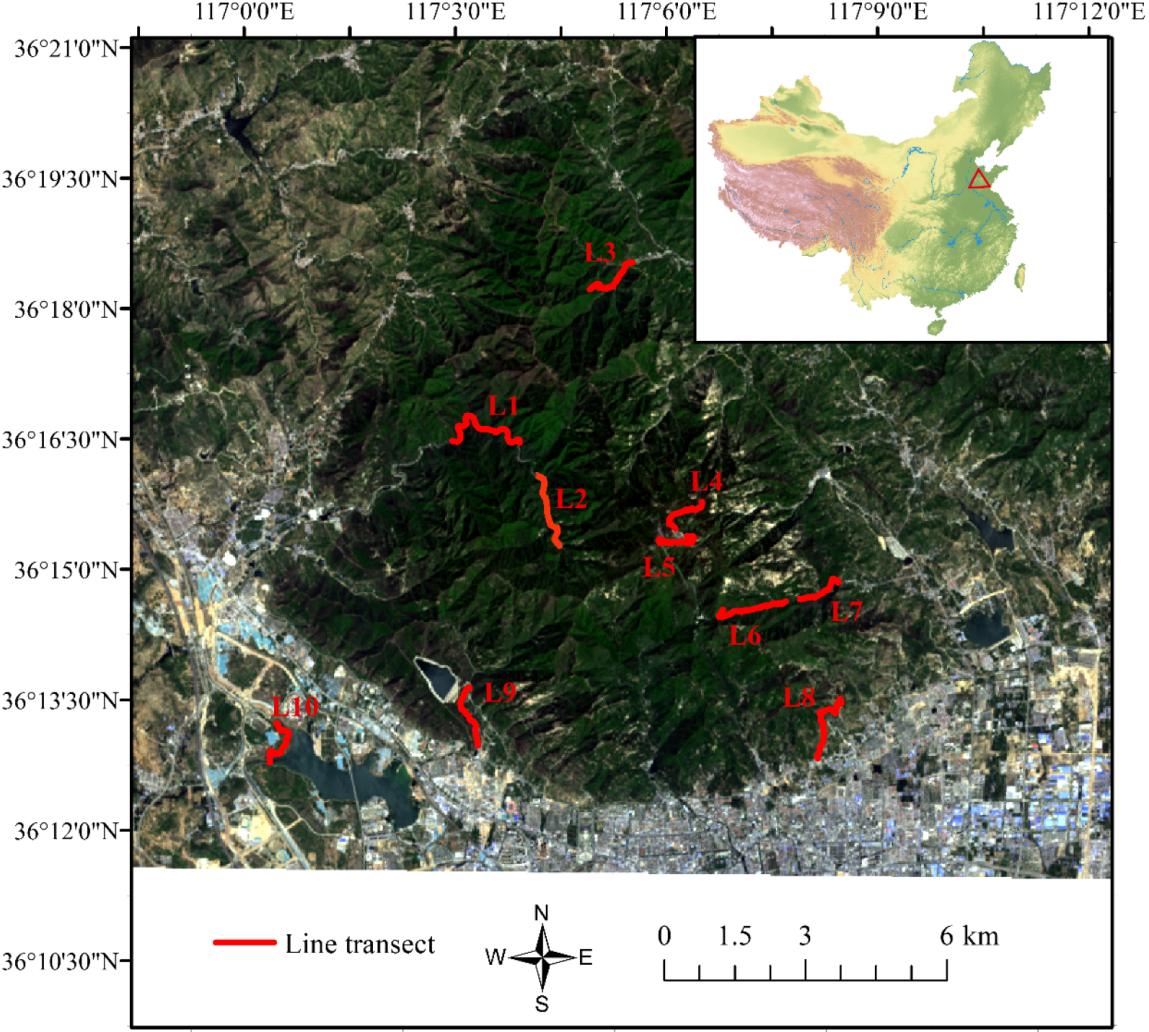
Survey line transects of birds in Mount Tai. Red triangles in the small map show the location of Mount Tai in China

In May or June from 2016 to 2019, ten line transects were used to monitor the breeding bird population in Mount Tai. Each transect is 1.5 km in length and 100 m in width. In breeding season of each year, this study organized professional investigators skilled in bird identification to conduct twice field surveys. The average speed of movement along the transect was 1–2 km/h, and the bird species, number of individuals, coordinates, distance to birds, habitat type, threat factors, and altitude were recorded. Detailed survey methods were based on “Technical guidelines for biodiversity monitoring – birds” (Standard number: HJ 710.4–2014) from Ministry of Ecology and Environment of the People's Republic of China (https://www.mee.gov.cn/ywgz/fgbz/bz/bzwb/stzl/201411/t20141106_291246.shtml). Ten line transects were designed to cover the majority of habitat types at different altitudes (L1 ~ L10, Figure 1) and detailed information of each line transects was shown in Table S2. L4 and L5 were set at high altitudes and composed mainly of forest. L2 was set at middle altitudes. L1, L3, L6, and L7 were set from low to middle altitudes and surrounded by forest and shrub. L8, L9, and L10 were set at low altitudes and composed mainly of shrub and construction.

### 2.3 Interpretation and environmental variables

**FIGURE 2:**
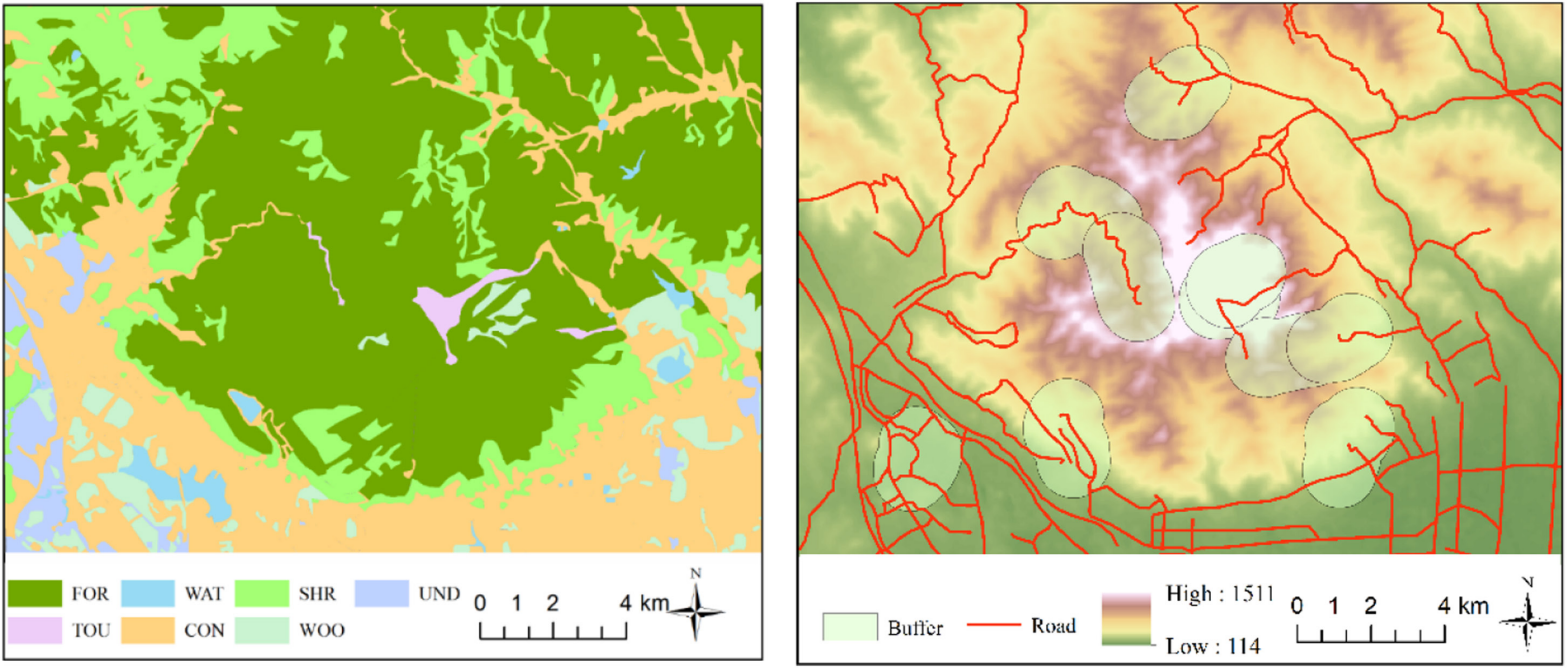
Land use (left) and landscape factors (right) in Mount Tai

A Landsat TM remote sensing image (May 20, 2019) of 30 m × 30 m resolution was downloaded with vertical direct sunlight angle to avoid the appearance of shadows in mountain. After field verification, we conducted the supervised interpretation of the remote sensing image to acquire the land use and cover using the software ENVI 5.1 (Figure 2). The land use in Mount Tai was divided into seven types: Forest (FOR), Shrub (SHR), Woodland (WOO), Water (WAT), Tourist area (TOU), Construction land (CON), and Undeveloped land (UND) (Table S1). If forest area with tree canopy density is equal or greater than 0.2, the land use is designated as Forest. If forest area with shrub coverage is equal or greater than 40%, the land use is designated as Shrubland. The woodland includes open forest land (forest area with tree canopy density ≥0.1 and<0.2), young afforested land, slash, and nursery garden. Continental water areas, ditches, and hydraulic structures were designated as Water. Artificial habitats were divided into three types based on the usages of land, Tourist area, Construction land, and Undeveloped land.

To quantify the habitat feature and landscape matrices, we generated oval spindle buffers around the line transects at a 1000 m as the radii. Compared to the area of Mount Tai, we defined 1000 m‐scale as the fine scales, then used ArcGIS 10.2 to extract road, altitude, and land use data within a 1000‐m buffer around every line transects (Figure 2). The WGS 1984 UTM zone 50 N was used as the uniform projected coordinate system. We conducted the analysis of the class matrix and landscape matrix using the software Fragstats 4.2 and then calculated the number of patches (NP), patch density (PD), and patch percentage of forest (FP, the percentage of the buffer covered by forest) within these buffers. The 8‐cell neighborhood rule was used as the operating parameter, and the sampling method was based on user‐provided points.

### 2.4 Vertical distribution index

Different bird species tended to be associated with different altitudes. We designed the Vertical Distribution Index (VDI) to indicate the selection of altitude for different species in Mount Tai. The numbers 1, 2, and 3 were the weights used for the percentage of the population of birds in the low‐altitude zone, middle‐altitude zone, and high‐altitude zone, respectively.

Vertical Distribution Index (VDI):

$$Y=\frac{n_{L}}{N}\times 1+\frac{n_{M}}{N}\times 2+\frac{n_{H}}{N}\times 3$$

VDI represents the Vertical Distribution Index of a given species; $n_{L}$ is the observed individual number of special birds in the low‐altitude zone; $n_{M}$ is the observed individual number of special birds in the middle‐altitude zone; $n_{H}$ is the observed individual number of special birds in the high‐altitude zone; *N* is the total observed individual number of special birds in Mount Tai. If the index approaches 1, the species tends to occur in the low‐altitude zone. If the index approaches 2, the species tends to occur in the middle‐altitude zone. If the index approaches 3, the species tends to occur in the high‐altitude zone. What is more, the index would approaches 2 as well if the species is uniformly distributed.

### 2.5 Phylogenetic diversity and functional diversity

To calculate phylogenetic diversity, we pruned the global phylogenetic tree of birds by subsampling 5000 Hackett All species: a set of 10,000 trees with 9993 OTUs each trees from BirdTree (http://birdtree.org; Jetz et al., 2012). Then we used the 5000 trees to construct a new maximum clade credibility tree with 0.5 posterior probability limit by the software TreeAnnotator v1.10.4 in the BEAST package. Based on the new phylogenetic tree constructed, we calculated Faith's PD to describe the total sum of phylogenetic history.

To calculate functional diversity, we chose 3 kinds of functional traits: morphological characteristics, feeding habits, and foraging strata (Wang et al., 2021; Wilman et al., 2014). We calculated phylogenetic signals using Blomberg's *K* for continuous traits (Blomberg et al., 2003) and statistic *D* for binary traits (Fritz & Purvis, 2010) to test trait conservatism. The R packages “phytools” and “caper” were used to calculate Blomberg's *K* (Revell, 2012) and statistic *D* (Orme et al., 2012), respectively. The results showed that phylogenetic signals were significantly related to most traits, indicating strong phylogenetic niche conservatism. Then, Gower dissimilarity was calculated from the species‐traits matrix. Based on Gower dissimilarity, we used the unweighted pair‐group method with arithmetic means (UPGMA) to construct a functional dendrogram, which was applied to calculate functional diversity (FD).

To infer whether the entire communities exhibited phylogenetic clustering or over‐dispersion, we calculated the standardized effect size (SES) of Faith's PD and FD. SES were calculated with R package “picante” (Kembel et al., 2010). SES indicate phylogenetic or functional clustering when <0, while over‐dispersion generates values higher than 0. One‐way ANOVA was used to test whether Faith's PD and FD were significantly different between three vertical belts. We used one‐sample t test to determine whether SES was significantly different from 0.

### 2.6 Statistic analysis

We calculated the total abundance, species richness, and Shannon‐Wiener index (*H′*) of per visit and transect to analyze variation in bird diversity using the Permute package, vegan package, and Spaa package. To examine the effect of landscape factors on bird diversity, we considered elevation, road, NP, PD, and FP as independent variables and the total abundance, species richness, and Shannon‐Wiener index (*H′*) of per visit and transect as dependent variables. Generalized linear model was used to analyze the linear relationship between landscape factors and bird diversity. Statistical relationships among independent variables and dependent variables were analyzed through Spearman's correlation analysis. All analyses were performed in the program R 3.5.1.

Multivariable linear regression analysis was also employed to analyze combined relationships between landscape factors and elevation. Richness and abundance of bird community were considered as dependent variables. Those factors were: elevation, distance to road, number of patches, patch density, and patch percentage. Min‐max normalization method was utilized to standardize all variables. After multicollinearity analysis, the number of patches was excluded. To examine the influence of remaining four factors on the bird richness and abundance, the weight and significance of different factors were determined. Multivariable linear regression analysis was operated in IBM SPSS version 22.0.

The percentage of bird taxonomic categories and areas in different land use types were determined. Based on the VDI and the number of each bird species in different land use types, we conducted a principal component analysis (PCA) on the different bird species. The two‐way cluster analysis and detrended correspondence analysis were conducted to analyze the relationship among different bird species along elevation using the Sorenson method in software PC‐ORD 6.0 (Wild Blueberry Media, LLC).

## 3 RESULTS

### 3.1 Changes in land use along the altitudinal gradient

The composition of land use varied with altitude (Table 1). The main type of land use was forest in the high‐mountain belt, accounting for 73.26% of the total area. There was also a small tourist area (24.68%). Forest was also the dominant landscape feature in the middle‐mountain belt, accounting for 87.69% of the total area. Forest, construction land, and shrub cover most of the low‐mountain belt, accounting for 88.84% of the total area. Compared with the high‐mountain belt and the middle‐mountain belt, there are more types of land use and areas in the low‐mountain belt, and the proportions of different types of land are more even. The area of forest is larger in the middle‐mountain belt than in the other belts. The area of construction land is the highest in the low‐mountain belt because of human activities and urbanization.

**TABLE 1:** Changes in land use types along an altitudinal gradient

No	Habitat types	Abbreviation	Percentage in mountain belt		
			High	Middle	Low
1	Forest	FOR	73.26%	87.69%	32.87%
2	Tourist area	TOU	24.68%	0.76%	0.10%
3	Water	WAT	0.00%	0.10%	1.82%
4	Construction land	CON	0.00%	1.33%	34.24%
5	Shrub	SHR	2.06%	9.15%	21.73%
6	Woodland	WOO	0.00%	0.98%	5.63%
7	Undeveloped land	UND	0.00%	0.00%	3.60%
	Total area (km^2^)		3.62	127.65	245.37

### 3.2 Bird community in different types of land use

**FIGURE 3:**
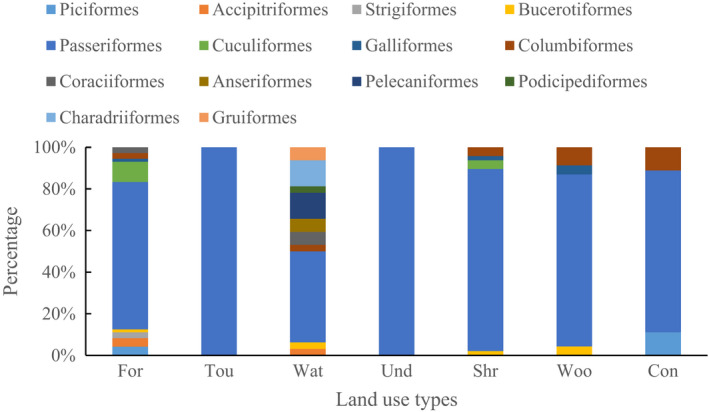
Bird community composition in different land use types. Abbreviations of land use types are defined in Table 1

From 2016 to 2019, 7444 birds from 113 species and 14 orders were detected in Mount Tai based on the IOC World Bird List Version 12.1 (Table S3). Species richness and abundance of birds were highest in forest with 72 species and 3117 individuals (Table S4). Whereas, artificial habitats, such as tourist area, construction land, and undeveloped land, supported lower richness and abundance of birds. Values of the Shannon‐Wiener index were higher in forest (3.29), water (2.75), and shrub (2.99) and lower in tourist area (0.12). The number of bird orders in water was greater compared with the other habitat types and included *Charadriiformes*, *Anseriformes*, and *Podicipediformes* (Figure 3). In construction, tourist area, and undeveloped land, *Passeriformes* and *Columbiformes* were the most common, including *Pycnonotus sinensis* and *Passer montanus*. Overall, *Passeriformes* was the main component of the bird community in Mount Tai.

### 3.3 Distribution pattern of bird diversity along an altitudinal gradient

**FIGURE 4:**
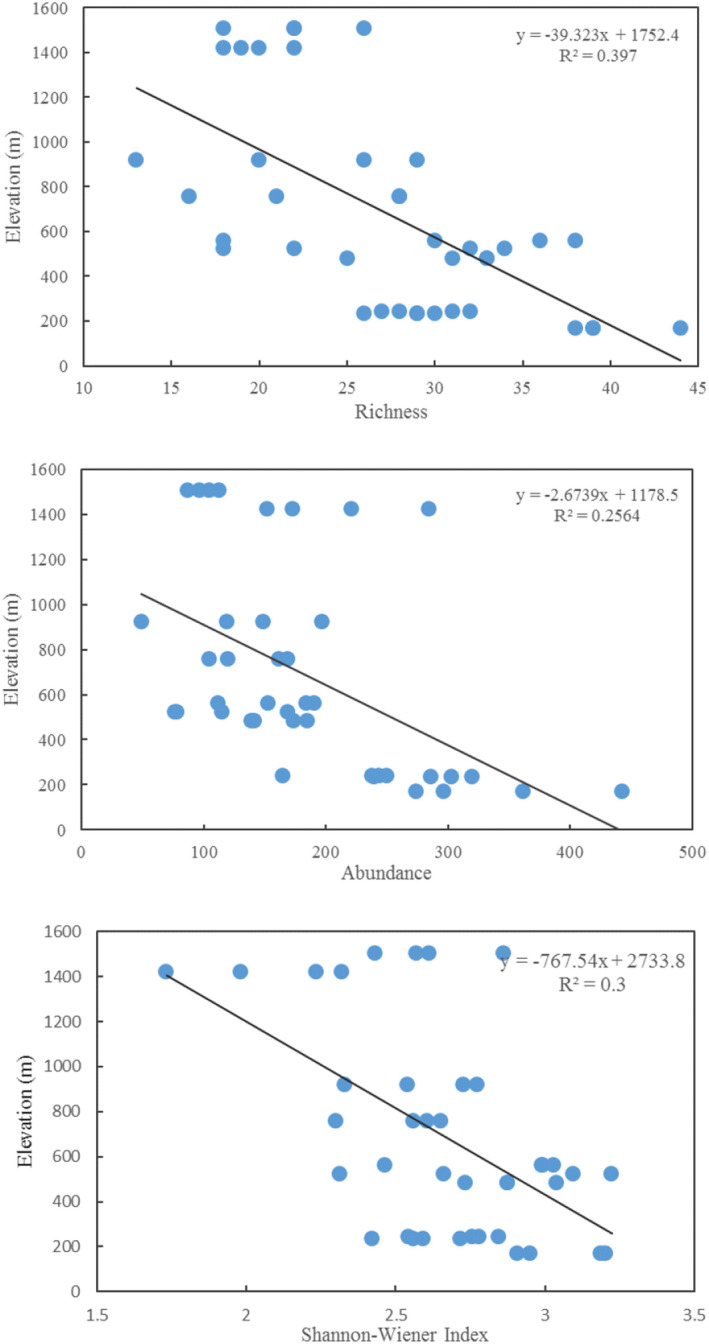
Changes in bird abundance, richness, and diversity with elevation in Mount Tai

**FIGURE 5:**
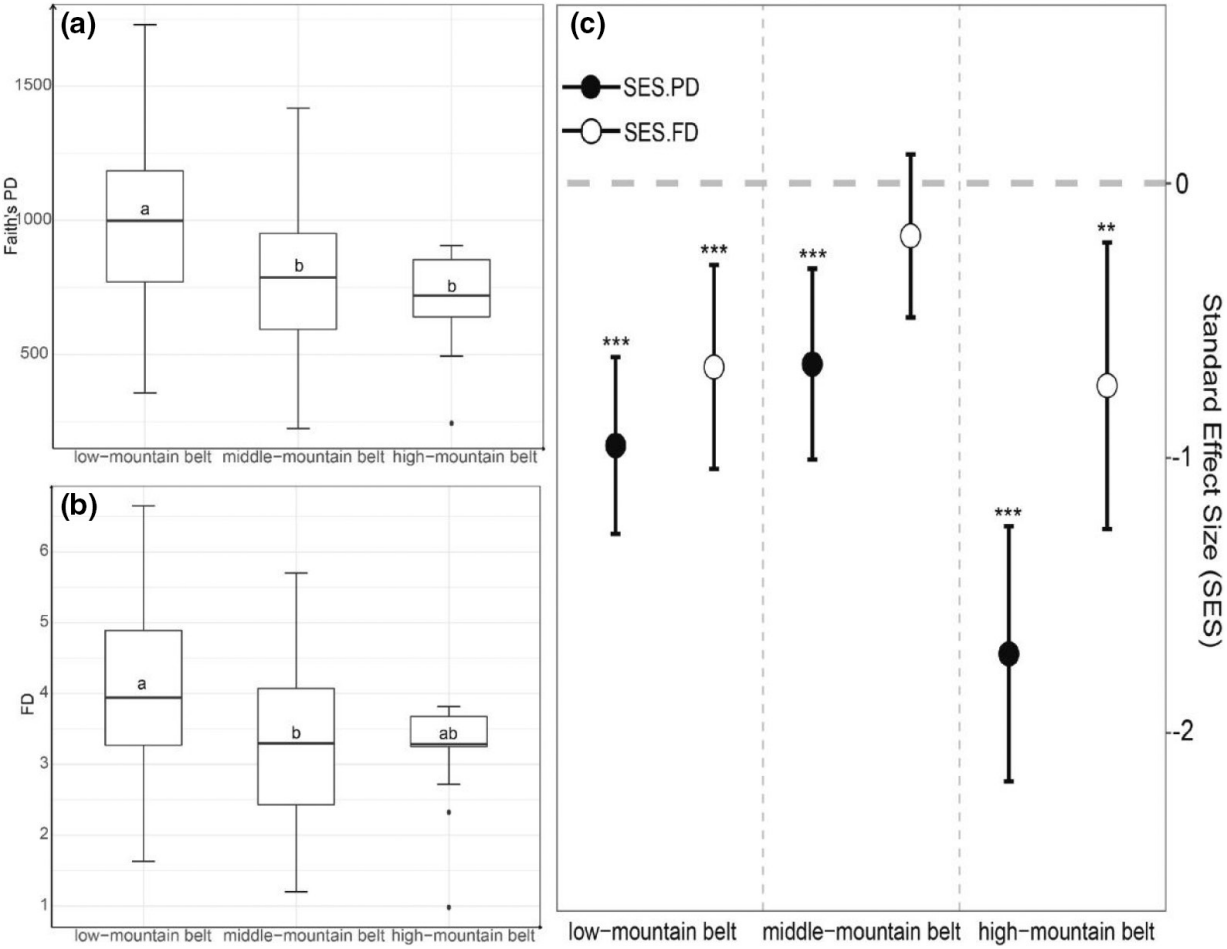
Phylogenetic diversity and functional diversity of bird communities in different vertical belts. (a) Faith's PD of bird communities in three vertical belts, with the same letter meaning no significant difference. (b) FD of bird communities in three vertical belts, with the same letter meaning no significant difference. (c) Standardized effect size (SES) of Faith's PD and FD of bird communities in three vertical belts. “***” means *p*‐value obtained from one‐sample t test is less than .001, “**” means *p*‐value is less than .01

In Mount Tai, the abundance, richness, and Shannon‐Wiener index of birds tended to decrease with altitude (Figure 4). The richness and abundance in low‐mountain belt was highest. Similarly, Faith's PD was significantly highest in low‐mountain belt (*F*_2,78_ = 7.10, *p* < .01), and there was significant difference in FD between low‐mountain belt and middle‐mountain belt (*F*_2,78_ = 4.66, *p* < .05) (Figure 5a,b). No significant difference was found in Faith's PD and FD between the middle‐mountain belt and the high‐mountain belt. SES.PD and SES.FD also showed a trend of increasing and then decreasing along with altitude gradient (Figure 5c).

**FIGURE 6:**
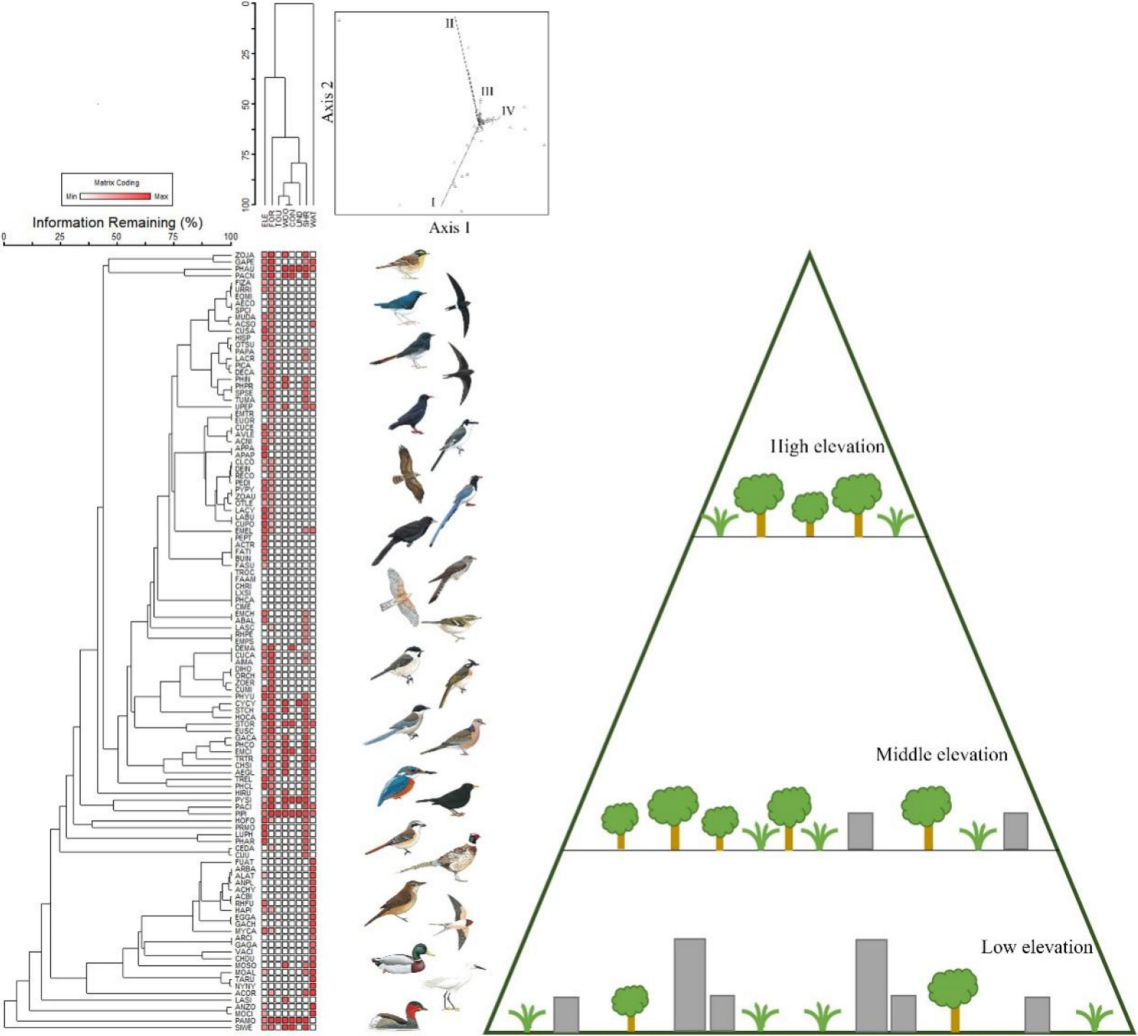
Vertical distribution pattern of the bird community in Mount Tai along the elevational gradient. Based on the land‐use pattern, elevation, and diversity, phylogenetic analysis indicated the relation among 113 bird species, PCA indicates four groups of birds in Mount Tai. Right figure shows the situation of land use along the elevation gradients roughly, including forest (tree), shrub (grass), and artificial habitat (gray histogram)

The relationships among different bird species were analyzed using a two‐way cluster analysis and detrended correspondence analysis (Figure 6). Diverse land use types leaded to divergent selection favoring different characteristics or activities of birds at different altitudes. Waterbirds were common in the low‐mountain belt, but raptor and some rare Passerines appeared in the middle or high mountain belt. Moreover, the value of SES.PD and SES.FD was almost smaller than 0, indicating phylogenetic and functional clustering of bird communities in three vertical belts (Figure 5c). SES.PD was significantly different from 0, and SES.FD was significantly different from 0 in low‐mountain belt and high‐mountain belt. There was no significant difference between SES.FD and 0 in middle‐mountain belt. Based on the features of birds at different altitudes, the structure of the bird community shows a fast turnover as bird diversity decreases with altitude. Moreover, it was also obvious that the landscape pattern have a huge change as elevation increases.

In the low‐mountain belt, construction land, water, and woodland supported some species from *Anseriformes*, *Columbiformes*, and *Passeriformes*, including *Tachybaptus ruficollis*, *Egretta garzetta*, *Streptopelia chinensis*, *Passer montanus*, and *Phasianus colchicus*. These bird species in the low‐mountain belt are highly adaptable to human activities and the city environment. In the middle‐mountain belt, some species that relied on forest and shrub were frequently observed, such as *Parus palustris*, *Urocissa erythrorhyncha*, and *Accipiter nisus*. These species primarily belonged to *Passeriformes* and *Accipitriformes*. In the high‐mountain belt, a large percentage of forest and shrub supported bird species with a preference for high elevation, such as *Luscinia phaenicuroides*, *Apus pacificus*, *Apus pacificus*, and *Prunella montanella*. What is more, the 113 bird species were clustered into four groups according to the PCA. Birds in forest formed Group 1, and birds in the other types of land use were closely related and formed Group II, III, and IV.

### 3.4 Effect of landscape factors on the vertical distribution of birds

**FIGURE 7:**
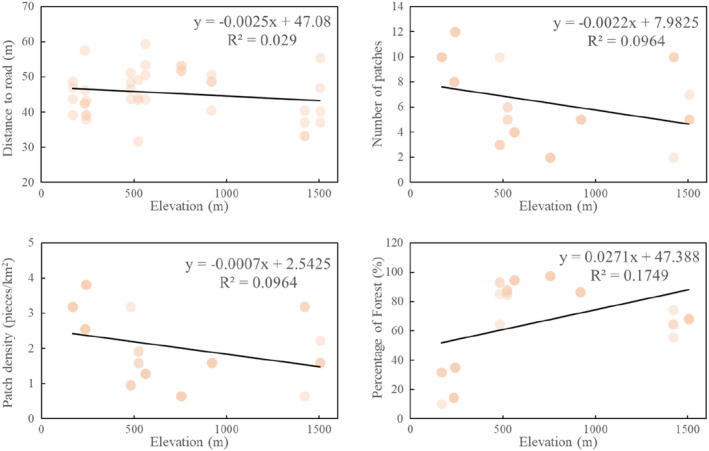
Variation in landscape factors with elevation

Number of patches (Spearman rank correlation, *r =* −0.43, *N =* 40, *p* < .05), and patch density (Spearman rank correlation, *r =* −0.45, *N =* 40, *p* < .05) had a significant negative correlation with elevation, and decreased as the altitude increased (Figure 7). However, the percentage of forest patches (Spearman rank correlation, *r =* 0.50, *N =* 40, *p* < .05) was positively related to elevation, and increased with increased elevation. Thus, the number of patches, patch density, and roads became more numerous at lower altitudes, which is where the degree of spatial fragmentation was higher.

**FIGURE 8:**
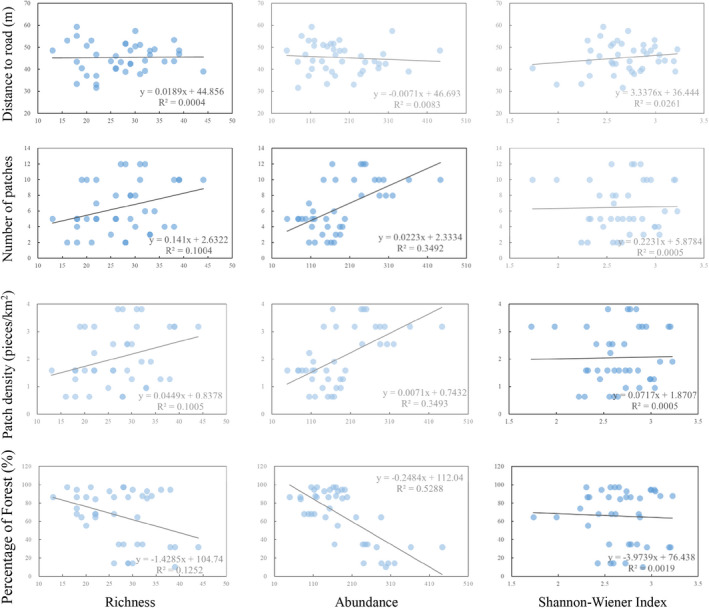
Effect of habitat factors on bird community characteristics

The correlation between four landscape factors and bird diversity was analyzed to characterize the landscape factors affecting the bird community (Figure 8). Bird richness and the diversity index increased steadily as the distance to road increases. The opposite pattern was observed for bird abundance. Distance to road has no noticeable effect on bird abundance (Spearman rank correlation, *r =* 0.04, *N =* 40, *p =* .80), richness (Spearman rank correlation, *r =* −0.14, *N =* 40, *p =* .38), and the Shannon‐Wiener index (Spearman rank correlation, *r =* 0.11, *N =* 40, *p =* .52). The increase in bird diversity was accompanied by an increase in the number of patches. The number of patches was significantly correlated with bird abundance (Spearman rank correlation, *r =* 0.52, *N =* 40, *p* < .05). Bird diversity increased with the patch density increases, including richness, abundance, and index. Bird richness (Spearman rank correlation, *r =* 0.34, *N =* 40, *p* < .05), and abundance (Spearman rank correlation, *r =* 0.55, *N =* 40, *p* < .05) were significantly positive correlated with patch density. However, there was a significant negative relation between the percentage of forest and bird abundance (Spearman rank correlation, *r =* −0.60, *N =* 40, *p* < .05).

Finally, we combined elevation with landscape factors to analyze the relationship between elevational variation and bird diversity under the background of spatial fragmentation. The equation of richness and four independent variables was “Richness = −0.414 × elevation + −0.034 × Distance to road + 0.082 × Patch density + −0.003 × Patch percentage + 0.601” (*R* ^2^ = 0.414) (Table 2). The equation of abundance and four independent variables was “Abundance = −0.149 × elevation + 0.042 × Distance to road + 0.052 × Patch density + −0.378 × Patch percentage + 0.602” (*R* ^2^ = 0.581). The result of multiple linear regression analysis indicated that elevation has a strong effect on bird richness a high weight of coefficient compared to others independent variables. Patch percentage had a high weight for bird abundance. The coefficient of distance to road and patch density was low in both two equations. Nevertheless, the coefficient of patch density is positive for bird richness and abundance.

**TABLE 2:** Regression analysis of the effect of landscape factors on bird abundance and richness

Dependent variable	Independent variable	Coefficient	*t*‐value	Significance
Richness	Elevation	−0.414	−3.983	0.001
	Distance to road	−0.034	−0.237	0.814
	Patch density	0.082	0.503	0.618
	Patch percentage	−0.003	−0.02	0.984
	Constant	0.601	3.119	0.004
Abundance	Elevation	−0.149	−1.822	0.077
	Distance to road	0.042	0.366	0.717
	Patch density	0.052	0.403	0.69
	Patch percentage	−0.378	−3.092	0.004
	Constant	0.602	3.978	0.001

## 4 DISCUSSION

### 4.1 Vertical distribution pattern of bird diversity

Based on continuous field survey, our results show Mount Tai harbors exceptionally high biodiversity, and supports many valuable and special species. This study demonstrated bird richness and abundance decreased with latitude and the composition of bird communities varied along altitudinal gradients. Though the distribution pattern is similar as some previous studies in other mountain ecosystem, the elevation–land use interactions are different and shape special mountain biodiversity in Mount Tai (Bellis et al., 2009; Kattan & Franco, 2004; McCain, 2009; Peters et al., 2019). Most studies examining the vertical distribution pattern of birds have focused on regions with natural habitat and with lower levels of disturbance. In some previous study mountain, these studies focused on the interaction of mountain substrates, life forms and climate system and described the relationships among diverse montane environments and biodiversity (Butchart et al., 2010; Colwell et al., 2004; Rahbek, Borregaard, Colwell, et al., 2019b). By contrast, fewer studies have examined the vertical distribution pattern of birds in regions near or inside cities (Pan et al., 2019; Ruggiero & Hawkins, 2008). By the field surveys in Gaoligong Mountains, studies indicated the necessity to promote conservation effort at the low elevation where is high richness of birds but intensive human land use (Pan et al., 2019).

Our findings suggest that there are more habitat types supporting higher diversity in the low‐mountain belt. Some species associated with urban areas are common in the low‐mountain belt, such as *Pycnonotus sinensis* and *Pica serica*. Abundant wetlands in the low‐mountain belt provide suitable habitat for waterbirds, such as *Tachybaptus ruficollis* and *Egretta garzetta*. In the middle‐mountain or high‐mountain belt, the richness and abundance of birds were lower, and the percentage of natural habitat was higher. Furthermore, a high percentage of forest and higher elevations are more suitable for some specialized species of *Passeriformes* and *Accipitriformes*, such as *Apus apus*, *Aviceda leuphotes*, *Luscinia phoenicuroides*, *Aviceda leuphotes*, *Phylloscopus armandii*, and *Rallina eurizonoides* (a new record for Shandong Province). There were significant distributional differences in the structure of the bird community at different altitudes in Mount Tai. The strong elevational shifts in habitat and topography often lead to strongly selective pressures in different belts (Loughnan & Gilbert, 2017). Phylogenetic diversity of bird communities decreases along with elevation gradient, which means the loss of phylogenetic history. In other words, subsets of phylogenetic clades may disappear in the high‐mountain belt. Presumably this is related to selection pressure in the high‐mountain belt. Birds could have a narrow distribution range in the high‐mountain belts of Mount Tain because of narrow physiological tolerances, life history characteristics, behavioral plasticity, and other factors (Jetz & Rahbek, 2002). The analysis of SES.PD also demonstrates that environmental filtering leads to phylogenetic clustering, especially in the high‐mountain belt where SES.PD is lowest. It reflects that selection pressure is highest in the high‐mountain belt. Generally, functional diversity of bird communities also decreases along with altitude gradient, which means that less niches are occupied by birds at high elevation gradient. Moreover, the highest SES.FD in middle‐mountain belt seems that environmental filtering has little effect on functional diversity in middle‐mountain belt. We think that vacated niches and competitive release are common in middle elevation gradient, leading to less stable functional structure.

The elevational pattern of bird diversity could be driven by multiple factors in Mount Tai surroundeded by cities and farms. Different from four possible patterns of variation in biodiversity along altitudinal gradients (decreasing diversity with increasing elevation; high diversity across a plateau of lower elevations, and then decreasing monotonically; a unimodal pattern with maximum diversity at intermediate elevations; increasing monotonically) (Mccain, 2007a; McCain, 2009; Quintero & Jetz, 2018), abiotic and biotic changes occur within short distances on mountainous gradients in Mount Tai. The elevational distribution pattern of bird diversity in Mount Tai is consistent with the first model (decreasing diversity with increasing elevation). The vertical distribution of bird diversity along altitudinal gradients is related to multiple factors, such as habitat area, temperature, human activities, and landscape (Colwell et al., 2004; Zhang et al., 2020). This finding implied that diversity is constrained in the high‐mountain belt and that there are abundant ecological niches in the middle and low mountain belt. Due to the rich landscape diversity, the patterns of habitat occupancy and distributional patterns contribute to enhancing biodiversity in the low‐mountain belt via the aggregation effect (Kattan & Franco, 2004).

### 4.2 Mechanism underlying the effects of multiple landscape factors

Mount Tai is a typical montane system that reflects the effect of human disturbance or spatial fragmentation on the vertical distribution pattern of biodiversity. Although Mount Tai is not as high as Tibet, there are diverse land types and different types of habitats. In Mount Tai, the level of development varies with altitude, and spatial fragmentation in the low‐mountain belt are relatively high because of extensive construction activities. The spatial disparity in spatial fragmentation is an unignorable reason for the vertical variation in the bird community. There are different human activities at various altitudes, and the bird community in middle and high‐altitude areas is also disturbed by human activities to a certain extent. Some landscape factors have a considerable influence on bird diversity and can be considered key factors, such as roads, real estate development, and tourism development (Pan et al., 2019; Peters et al., 2019).

The degree of human disturbance in the low‐mountain belt is higher than other belts, and the elevational pattern of bird diversity is consistent with the variance of landscape pattern. Though most of the habitats were artificial surfaces and with high human disturbance, the low elevational bands of Mount Tai harbored a larger area and there were more habitat types. A large surface area and wide range of available habitats could facilitate the establishment of immigrant lineages from surrounding lowlands and other mountains, further increasing species richness (Pan et al., 2019). What is more, Over millions of years, land use in montane systems often experiences large changes as natural habitats are continuously converted to other land types, including roads, towns, tourist areas, and parks. These processes generally lead to a concentration of species at low to middle elevations, and a decrease in species richness toward mountain peaks (Antonelli et al., 2018; McCain & Grytnes, 2010). As in most regions in the Hengduan Mountains, most of the cities, villages, and farms in Mount Tai were distributed at low elevations and have been for thousands of years. This long history of disturbance may have exacerbated the evolution and adaptation of biodiversity (Pan et al., 2019; Wambulwa et al., 2021).

Many hypotheses have been proposed to explain elevational distribution patterns of biodiversity. However, mountains surrounding cities have generally been neglected, and most studies do not carefully consider the effect of landscape factors (Kattan & Franco, 2004; Wu et al., 2010). Spatial fragmentation caused by fine‐scale and diverse habitats play a vital role in biodiversity and ecosystem health (Pimm et al., 2014; Wilson et al., 2016). Though the result of multiple linear regression analysis demonstrates that elevation have a high effect on bird diversity, the landscape factors have a certain weight. Because of human activity and urbanization, the connectivity and quality of habitat have been greatly compromised (Haddad et al., 2015). There is thus a need to identify the effect of spatial fragmentation on the elevational distribution pattern of birds in mountainous ecosystems faced with urbanization. The “species‐area relationship” describing the regular pattern between species abundance and habitat area is one of the core hypotheses of montane ecology (Dengler, 2009; Losos & Schluter, 2000). There is a negative correlation between bird diversity and the patch area of forest, indicating that larger areas of natural habitat correspond to lower bird diversity. This relationship is not consistent with the “species‐area relationship.” Similarly, the analysis of 150 bird datasets of montane systems revealed that the “species‐area relationship” might often not be suitable for explaining the vertical distribution pattern of bird diversity simply (McCain, 2009). The number of patches and patch density are important indicators reflecting spatial fragmentation (Haddad et al., 2015). Compared with previous montane studies, this study found that bird diversity was positively correlated with the number of patches and patch density. However, the number of patches and patch density were negatively correlated with altitude. To make sense of the effects of spatial fragmentation on species richness, the “habitat amount hypothesis” was recently proposed, which notes that what is important is the total amount of habitat in an appropriate spatial extent of the local landscape independent of its spatial configuration (Fahrig, 2013; Fahrig et al., 2019; Rybicki et al., 2020). When the area of habitat is larger, habitat fragmentation may increase species diversity. There is more diverse landscape pattern in low‐mountain belt, and the total amount of habitat in Mount Tai is sufficient. The spatial fragmentation in the low‐belt mountain with sufficient area is higher than the high‐belt mountain and the richness and abundance of birds community in the low‐belt mountain is relatively higher. Thus, the “habitat amount hypothesis” may provide a better explanation for the elevational distribution pattern in Mount Tai.

### 4.3 Land management and biodiversity conservation

Colorful biological resources in Mount Tai are important for the well‐being of the people and the maintenance of biodiversity. If lack of suitable land management and protective action, high‐intensity tourism development, engineering construction, and human activities could seriously affect the diversity of birds along elevational gradients. Therefore, we propose four suggestions: First, ecological tourism is urgently developed to promote the combination of ecological balance and nature protection. Sustainable tourism, which differs from other kinds of tourism, will do no harm to environment. Second, continuous biodiversity monitoring involving climate, soil, vegetation, and birds is meaningful for us to have a complete understanding of dynamic change in biodiversity. In addition, reasonable evaluation indexes could alert the land degradation and the loss of biodiversity. Third, it has a very strong practical significance to conduct ecological restoration under different natural conditions and human activities. For example, the Taohuayu mountaineering route, which is used to reduce stress from many passengers, was damaged by high‐intensity human activities and the landscape pattern is single because of the artificial landscape. Repairing infrastructure, planting native plants, setting up signs were suggested strongly. For some areas with serious habitat degradation, closed management should be carried out. Fourth, rich natural resources should be sufficiently utilized to raise ecological awareness. Community, universities, institutes, and government should be connected closely. Public events also should be organized regularly to promote the dissemination of scientific ideas.

## 5 CONCLUSION

The Montane forest is an important ecosystem to maintain high biodiversity. Due to the rapid development of urbanization and human activity, these patterns of biodiversity in different mountainous belts are suffering various levels of artificial disturbance except elevation. This work integrated bird diversity with landscape factors to reveal the effects of spatial fragmentation on the vertical distribution pattern at different altitudes. Our results demonstrated bird diversity decreased as the altitude increased, and the percentage of different land types and the structure of the bird community significantly differed in the different belts. Special habitats with harsh environment have a constraint on the structure of bird community and some rare species tended to only occupy specific belts. Moreover, our study indicated sufficient habitat and more patches in the low‐mountain belt supported higher bird diversity. The “habitat amount hypothesis” was more suitable for explaining the elevational distribution pattern of bird diversity in a typical modern mountain. Our results highlight the effects of ongoing urbanization and human activities on montane biodiversity and emphasize the management of artificial habitats.

## AUTHOR CONTRIBUTIONS

**Wei Liu:** Conceptualization (equal); formal analysis (equal); software (equal); validation (equal); writing – original draft (equal); writing – review and editing (equal). **Dandan Yu:** Methodology (equal); software (equal). **Sijia Yuan:** Methodology (supporting); writing – review and editing (equal). **Jianfeng Yi:** Formal analysis (equal); software (equal). **Yun Cao:** Supervision (equal); writing – original draft (equal). **Xiufen Li:** Data curation (equal); resources (equal). **Haigen Xu:** Conceptualization (equal); funding acquisition (equal); writing – review and editing (equal).

## CONFLICT OF INTEREST

All authors state that there is no conflict of interest.

## Supporting information


**TABLE S1**Land use and land cover (LULC) types

## Data Availability

All data have been archived and made available at https://doi.org/10.5061/dryad.866t1g1q2.

## References

[ece39051-bib-0001] Antonelli, A. , Kissling, W. D. , Flantua, S. G. , Bermúdez, M. A. , Mulch, A. , Muellner‐Riehl, A. N. , Kreft, H. , Linder, P. , Badgley, C. , Fjeldsa, J. , Fritz, S. A. , Rahbek, C. , Herman, F. , Hoogiemstra, H. , & Hoorn, C. (2018). Geological and climatic influences on mountain biodiversity. Nature Geoscience, 11(10), 718–725.

[ece39051-bib-0002] Barbier, E. B. , Burgess, J. C. , & Dean, T. J. (2018). How to pay for saving biodiversity. Science, 360(6388), 486–488. 29724939 10.1126/science.aar3454

[ece39051-bib-0003] Bellis, L. M. , Rivera, L. , Politi, N. , Martín, E. , Perasso, M. L. , Cornell, F. , & Renison, D. (2009). Latitudinal patterns of bird richness, diversity and abundance in Polylepis australis mountain forest of Argentina. Bird Conservation International, 19(3), 265–276.

[ece39051-bib-0004] Blomberg, S. P. , Garland, T., Jr. , & Ives, A. R. (2003). Testing for phylogenetic signal in comparative data: Behavioral traits are more labile. Evolution, 57(4), 717–745. 12778543 10.1111/j.0014-3820.2003.tb00285.x

[ece39051-bib-0005] Butchart, S. H. , Walpole, M. , Collen, B. , Van, S. A. , Scharlemann, J. P. , Almond, R. E. , Baillie, J. E. , Bomhard, B. , Brown, C. , & Bruno, J. (2010). Global biodiversity: Indicators of recent declines. Science, 328(5982), 1164–1168. 20430971 10.1126/science.1187512

[ece39051-bib-0006] Colwell, R. K. , Rahbek, C. , & Gotelli, N. J. (2004). The mid‐domain effect and species richness patterns: What have we learned so far? The American Naturalist, 163(3), 1–23. 10.1086/382056 15026983

[ece39051-bib-0007] Dengler, J. (2009). Which function describes the species–area relationship best? A review and empirical evaluation. Journal of Biogeography, 36(4), 728–744.

[ece39051-bib-0008] Du, H. (1985). The vertical distribution of birds on Taishan mountain. Sichuan Journal of Zoology, 4(1), 9–13.

[ece39051-bib-0009] Emel, S. L. , Wang, S. , Metz, R. P. , & Spigler, R. B. (2021). Type and intensity of surrounding human land use, not local environment, shape genetic structure of a native grassland plant. Molecular Ecology, 30(3), 639–655. 33245827 10.1111/mec.15753

[ece39051-bib-0010] Fahrig, L. (2003). Effects of habitat fragmentation on biodiversity. Annual Review of Ecology, Evolution, and Systematics, 34(1), 487–515.

[ece39051-bib-0011] Fahrig, L. (2013). Rethinking patch size and isolation effects: The habitat amount hypothesis. Journal of Biogeography, 40(9), 1649–1663.

[ece39051-bib-0012] Fahrig, L. (2017). Ecological responses to habitat fragmentation per se. Annual Review of Ecology, Evolution, 48(1), 1–23.

[ece39051-bib-0013] Fahrig, L. , Arroyo‐Rodríguez, V. , Bennett, J. R. , Boucher‐Lalonde, V. , Cazetta, E. , Currie, D. J. , Eigenbrod, F. , Ford, A. T. , Harrison, S. P. , Jaeger, J. A. G. , Koper, N. , Martin, A. E. , Martin, J. L. , Metzger, J. P. , Morrison, P. , Rhodes, J. R. , Saunders, D. A. , Simberloff, D. , Smith, A. C. , … Watling, J. I. (2019). Is habitat fragmentation bad for biodiversity? Biological Conservation, 230(1), 179–186.

[ece39051-bib-0014] Fletcher, R. J. , Reichert, B. E. , & Holmes, K. (2018). The negative effects of habitat fragmentation operate at the scale of dispersal. Ecology, 99(10), 2176–2186. 30112822 10.1002/ecy.2467

[ece39051-bib-0015] Fritz, S. A. , & Purvis, A. (2010). Selectivity in mammalian extinction risk and threat types: A new measure of phylogenetic signal strength in binary traits. Conservation Biology, 24(4), 1042–1051. 20184650 10.1111/j.1523-1739.2010.01455.x

[ece39051-bib-0016] Gao, J. , Liu, Y. , & Bogonovich, M. (2018). Habitat is more important than climate and animal richness at shaping latitudinal variation in plant diversity in China. Biodiversity and Conservation, 27(14), 3679–3691.

[ece39051-bib-0017] García‐Llamas, P. , Calvo, L. , De la Cruz, M. , & Suárez‐Seoane, S. (2018). Landscape heterogeneity as a surrogate of biodiversity in mountain systems: What is the most appropriate spatial analytical unit? Ecological Indicators, 85, 285–294.

[ece39051-bib-0018] Haddad, N. M. , Brudvig, L. A. , Clobert, J. , Davies, K. F. , Gonzalez, A. , Holt, R. D. , Lovejoy, T. E. , Sexton, J. O. , Austin, M. P. , Collins, C. D. , Cook, W. M. , Damschen, E. I. , Ewers, R. M. , Foster, B. L. , Jenkins, C. N. , King, A. J. , Laurance, W. F. , Levey, D. J. , Margules, C. R. , … Townshend, J. R. (2015). Habitat fragmentation and its lasting impact on Earth's ecosystems. Science Advances, 1(2), e1500052. 26601154 10.1126/sciadv.1500052 PMC4643828

[ece39051-bib-0019] Harris, G. , & Pimm, S. L. (2010). Range size and extinction risk in Forest birds. Conservation Biology, 22(1), 163–171. 10.1111/j.1523-1739.2007.00798.x 18254861

[ece39051-bib-0020] Hu, C. , Yuan, S. , Sun, W. , Chen, W. , Liu, W. , Li, P. , & Chang, Q. (2021). Spatial genetic structure and demographic history of the wild boar in the Qinling Mountains, China. Animals, 11(2), 346. 33572967 10.3390/ani11020346 PMC7912324

[ece39051-bib-0021] Jetz, W. , & Rahbek, C. (2002). Geographic range size and determinants of avian species richness. Science, 297(5586), 1548–1551. 12202829 10.1126/science.1072779

[ece39051-bib-0022] Jetz, W. , Thomas, G. H. , Joy, J. B. , Hartmann, K. , & Mooers, A. O. (2012). The global diversity of birds in space and time. Nature, 491(7424), 444–448. 23123857 10.1038/nature11631

[ece39051-bib-0023] Jetz, W. , Wilcove, D. S. , & Dobson, A. P. (2007). Projected impacts of climate and land‐use change on the global diversity of birds. PLoS Biology, 5(6), e157. 17550306 10.1371/journal.pbio.0050157 PMC1885834

[ece39051-bib-0025] Katayama, N. , Amano, T. , Naoe, S. , Yamakita, T. , Komatsu, I. , Takagawa, S. I. , Sato, N. , Ueta, M. , & Miyashita, T. (2014). Landscape heterogeneity–biodiversity relationship: Effect of range size. PLoS One, 9(3), e93359. 24675969 10.1371/journal.pone.0093359 PMC3968173

[ece39051-bib-0026] Kattan, G. H. , & Franco, P. (2004). Bird diversity along elevational gradients in the Andes of Colombia: Area and mass effects. Global Ecology and Biogeography, 13(5), 451–458.

[ece39051-bib-0027] Kembel, S. W. , Cowan, P. D. , Helmus, M. R. , Cornwell, W. K. , Morlon, H. , Ackerly, D. D. , Blomberg, S. P. , & Webb, C. O. (2010). Picante: R tools for integrating phylogenies and ecology. Bioinformatics, 26(11), 1463–1464. 20395285 10.1093/bioinformatics/btq166

[ece39051-bib-0028] Lele, A. , Arasumani, M. , Vishnudas, C. K. , Joshi, V. , Jathanna, D. , & Robin, V. V. (2020). Elevation and landscape change drive the distribution of a montane, endemic grassland bird. Ecology and Evolution, 10(14), 7755–7767. 32760562 10.1002/ece3.6500 PMC7391316

[ece39051-bib-0029] Losos, J. B. , & Schluter, D. (2000). Analysis of an evolutionary species–area relationship. Nature, 408(6814), 847–850. 11130721 10.1038/35048558

[ece39051-bib-0030] Loughnan, D. , & Gilbert, B. (2017). Trait‐mediated community assembly: Distinguishing the signatures of biotic and abiotic filters. Oikos, 126(8), 1112–1122.

[ece39051-bib-0031] Mittelbach, G. G. , Schemske, D. W. , Cornell, H. V. , Allen, A. P. , Brown, J. M. , Bush, M. B. , Harrison, S. P. , Hurlbert, A. H. , Knowlton, N. , Lessios, H. A. , McCain, C. M. , McCune, A. R. , McDade, L. A. , McPeek, M. A. , Near, T. J. , Price, T. D. , Ricklefs, R. E. , Roy, K. , Sax, D. F. , … Turelli, M. (2010). Evolution and the latitudinal diversity gradient: Speciation, extinction and biogeography. Ecology Letters, 10(4), 315–331. 10.1111/j.1461-0248.2007.01020.x 17355570

[ece39051-bib-0032] McCain, C. M. (2005). Elevational gradients in diversity of small mammals. Ecology, 86(2), 366–372.

[ece39051-bib-0033] McCain, C. M. (2007a). Area and mammalian elevational diversity. Ecology, 88(1), 76–86a. 17489456 10.1890/0012-9658(2007)88[76:aamed]2.0.co;2

[ece39051-bib-0034] McCain, C. M. (2007b). Could temperature and water availability drive elevational species richness patterns? A global case study for bats. Global Ecology and Biogeography, 16(1), 1–13b.

[ece39051-bib-0035] McCain, C. M. (2009). Global analysis of bird elevational diversity. Global Ecology and Biogeography, 18(3), 346–360.

[ece39051-bib-0036] McCain, C. M. , & Grytnes, J. A. (2010). Elevational gradients in species richness. Encyclopedia of Life Sciences. John Wiley & Sons, Ltd.

[ece39051-bib-0037] Orme, D. , Freckleton, R. , Thomas, G. , Petzoldt, T. , Fritz, S. , Isaac, N. , & Pearse, W. (2012). Caper: comparative analyses of phylogenetics and evolution in R. R package version 0.5.2, 458.

[ece39051-bib-0038] Pan, X. , Liang, D. , Zeng, W. , Hu, Y. , Liang, J. , Wang, X. , Robinson, S. , Luo, X. , & Liu, Y. (2019). Climate, human disturbance and geometric constraints drive the elevational richness pattern of birds in a biodiversity hotspot in Southwest China. Global Ecology and Conservation, 18, e00630.

[ece39051-bib-0039] Peh, K. S. H. , de Jong, J. , Sodhi, N. S. , Lim, S. L. H. , & Yap, C. A. M. (2005). Lowland rainforest avifauna and human disturbance: Persistence of primary forest birds in selectively logged forests and mixed‐rural habitats of southern peninsular Malaysia. Biological Conservation, 123(4), 489–505.

[ece39051-bib-0040] Pereira, H. M. , Leadley, P. W. , Proença, V. , Alkemade, R. , Scharlemann, J. P. , Fernandez‐Manjarrés, J. F. , Araujo, M. B. , Balvanera, P. , Biggs, R. , Cheung, W. W. L. , Chini, L. , Cooper, H. D. , Gilman, E. L. , Guénette, S. , Hurtt, G. C. , Huntington, H. P. , Mace, G. M. , Oberdorff, T. , Revenga, C. , … Walpole, M. (2010). Scenarios for global biodiversity in the 21st century. Science, 330(6010), 1496–1501. 20978282 10.1126/science.1196624

[ece39051-bib-0041] Peters, M. K. , Hemp, A. , Appelhans, T. , Becker, J. N. , Behler, C. , Classen, A. , . Detsch, F. , Ensslin, A. , Ferger, S. W. , Frederiksen, S. B. , Gebert, F. , Gerschlauer, F. , Gütlein, A. , Helbig‐Bonitz, M. , Hemp, C. , Kindeketa, W. J. , Kühnel, A. , Mayr, A. V. , Mwangomo, E. , … Steffan‐Dewenter, I. (2019). Climate–land‐use interactions shape tropical mountain biodiversity and ecosystem functions. Nature, 568(7750), 88–92. 30918402 10.1038/s41586-019-1048-z

[ece39051-bib-0042] Pimm, S. L. , Jenkins, C. N. , Abell, R. , Brooks, T. M. , Gittleman, J. L. , Joppa, L. N. , Raven, P. H. , Roberts, C. M. , & Sexton, J. O. (2014). The biodiversity of species and their rates of extinction, distribution, and protection. Science, 344(6187), 987–987. 10.1126/science.1246752 24876501

[ece39051-bib-0043] Pounds, J. A. , Bustamante, M. R. , Coloma, L. A. , Consuegra, J. A. , Fogden, M. P. , Foster, P. N. , Marca, E. L. , Masters, K. L. , Merino‐Viteri, A. , Puschendorf, R. , Ron, S. S. , Sánchez‐Azofeifa, G. A. , Still, C. J. , & Young, B. E. (2006). Widespread amphibian extinctions from epidemic disease driven by global warming. Nature, 439(7073), 161–167. 16407945 10.1038/nature04246

[ece39051-bib-0044] Pounds, J. A. , Fogden, M. P. , & Campbell, J. H. (1999). Biological response to climate change on a tropical mountain. Nature, 398(6728), 611–615.

[ece39051-bib-0045] Quintero, I. , & Jetz, W. (2018). Global elevational diversity and diversification of birds. Nature, 555(7695), 246–250. 29466335 10.1038/nature25794

[ece39051-bib-0046] Rahbek, C. , Borregaard, M. K. , Antonelli, A. , Colwell, R. K. , Holt, B. G. , Nogues‐Bravo, D. , Rasmussen, C. M. , Richardson, K. , Rosing, M. T. , Whittaker, R. J. , & Fjeldsa, J. (2019a). Building mountain biodiversity: Geological and evolutionary processes. Science, 365(6458), 1114–1119. 31515384 10.1126/science.aax0151

[ece39051-bib-0047] Rahbek, C. , Borregaard, M. K. , Colwell, R. K. , Dalsgaard, B. , Holt, B. G. , Morueta‐Holme, N. , Nogues‐Bravo, D. , Whittaker, R. J. , & Fjeldsa, J. (2019b). Humboldt's enigma: What causes global patterns of mountain biodiversity? Science, 365(6458), 1108–1113. 31515383 10.1126/science.aax0149

[ece39051-bib-0048] Revell, L. J. (2012). Phytools: An R package for phylogenetic comparative biology (and other things). Methods in Ecology and Evolution, 2, 217–223.

[ece39051-bib-0049] Ruggiero, A. , & Hawkins, B. A. (2008). Why do mountains support so many species of birds? Ecography, 31(3), 306–315.

[ece39051-bib-0050] Rybicki, J. , Abrego, N. , & Ovaskainen, O. (2020). Habitat fragmentation and species diversity in competitive communities. Ecology Letters, 23(3), 506–517. 31863571 10.1111/ele.13450 PMC7027465

[ece39051-bib-0051] Soh, M. C. , Sodhi, N. S. , & Lim, S. L. (2006). High sensitivity of montane bird communities to habitat disturbance in peninsular Malaysia. Biological Conservation, 129(2), 149–166.

[ece39051-bib-0052] Song, S. , Liu, X. , Bai, X. , Jiang, Y. , Zhang, X. , Yu, C. , & Shao, X. (2015). Impacts of environmental heterogeneity on moss diversity and distribution of Didymodon (Pottiaceae) in Tibet, China. PLos One, 10(7), e0132346. 26181326 10.1371/journal.pone.0132346 PMC4504491

[ece39051-bib-0053] Spehn, E. M. , Rudmann‐Maurer, K. , & Körner, C. (2011). Mountain biodiversity. Plant Ecology & Diversity, 4(4), 301–302.

[ece39051-bib-0054] Steinbauer, M. J. , Grytnes, J. A. , Jurasinski, G. , Kulonen, A. , Lenoir, J. , Pauli, H. , Rixen, C. , Winkler, M. , Bardy‐Durchhalter, M. , Barni, E. , Bjokman, A. D. , Breiner, F. T. , Burg, S. , Czortek, P. , Dawes, M. A. , Delimat, A. , Dullinger, S. , Erschbamer, B. , Felde, V. A. , … Wipf, S. (2018). Accelerated increase in plant species richness on mountain summits is linked to warming. Nature, 556(7700), 231–234. 29618821 10.1038/s41586-018-0005-6

[ece39051-bib-0055] Tabarelli, M. , Mantovani, W. , & Peres, C. A. (1999). Effects of habitat fragmentation on plant guild structure in the montane Atlantic forest of southeastern Brazil. Biological Conservation, 91(2–3), 119–127.

[ece39051-bib-0056] Terborgh, J. , Robinson, S. K. , Parker, T. A., III , Munn, C. A. , & Pierpont, N. (1990). Structure and organization of an Amazonian forest bird community. Ecological Monographs, 60(2), 213–238.

[ece39051-bib-0057] Wambulwa, M. C. , Milne, R. , Wu, Z. Y. , Spicer, R. A. , Provan, J. , Luo, Y. H. , Zhu, G. F. , Wang, W. T. , Wang, H. , Gao, L. M. , Li, D. Z. , & Liu, J. (2021). Spatiotemporal maintenance of flora in the Himalaya biodiversity hotspot: Current knowledge and future perspectives. Ecology and Evolution, 11(16), 10794–10812. 34429882 10.1002/ece3.7906 PMC8366862

[ece39051-bib-0058] Wang, X. , & Li, C. (2013). The biodiversity of Taishan Mountain. Intellectual Property Publishing House.

[ece39051-bib-0059] Wang, Y. , Song, Y. , Zhong, Y. , Chen, C. , Zhao, Y. , Zeng, D. , Wu, Y. , & Ding, P. (2021). A dataset on the life‐history and ecological traits of Chinese birds. Biodiversity Science, 29(9), 1149–1153.

[ece39051-bib-0060] Wilman, H. , Belmaker, J. , Simpson, J. , de la Rosa, C. , Rivadeneira, M. M. , & Jetz, W. (2014). EltonTraits 1.0: Species level foraging attributes of the world's birds and mammals: Ecological archives E095 178. Ecology, 95(7), 2027–2027.

[ece39051-bib-0061] Wilson, M. C. , Chen, X. Y. , Corlett, R. T. , Didham, R. K. , Ding, P. , Holt, R. D. , Holyoak, M. , Hu, G. , Hughes, A. C. , Jiang, L. , Laurance, W. F. , Liu, J. J. , Pimm, S. L. , Robinson, S. K. , Russo, S. E. , Si, X. F. , Wilcove, D. S. , Wu, J. G. , & Yu, M. J. (2016). Habitat fragmentation and biodiversity conservation: Key findings and future challenges. Landscape Ecology, 31(1), 219–227.

[ece39051-bib-0062] Woodbridge, J. , Fyfe, R. , Smith, D. , Pelling, R. , de Vareilles, A. , Batchelor, R. , Bevan, A. , & Davies, A. L. (2021). What drives biodiversity patterns? Using long‐term multidisciplinary data to discern centennial‐scale change. Journal of Ecology, 109(3), 1396–1410.

[ece39051-bib-0063] Wu, F. , Yang, X. J. , & Yang, J. X. (2010). Additive diversity partitioning as a guide to regional montane reserve design in Asia: An example from Yunnan Province, China. Diversity and Distributions, 16(6), 1022–1033.

[ece39051-bib-0064] Xiao, Y. S. , & Luo, G. (2005). Landscape structures and fractal analyses of Taishan Mountain, Shandong Province. Acta Ecologica Sinica, 25(1), 129–134.

[ece39051-bib-0065] Xu, H. , Cao, Y. , Yu, D. , Cao, M. , He, Y. , Gill, M. , & Pereira, H. M. (2021). Ensuring effective implementation of the post‐2020 global biodiversity targets. Nature Ecology & Evolution, 5(4), 411–418. 33495589 10.1038/s41559-020-01375-y

[ece39051-bib-0066] Xu, X. , Xie, Y. , Qi, K. , Luo, Z. , & Wang, X. (2018). Detecting the response of bird communities and biodiversity to habitat loss and fragmentation due to urbanization. Science of the Total Environment, 624, 1561–1576. 29929265 10.1016/j.scitotenv.2017.12.143

[ece39051-bib-0067] Zhang, Q. , Holyoak, M. , Chen, C. , Liu, Z. , Liu, J. , Che, X. , Dong, A. , Yang, C. , & Zou, F. (2020). Trait‐mediated filtering drives contrasting patterns of species richness and functional diversity across montane bird assemblages. Journal of Biogeography, 47(1), 301–312.

