# Supplementary material for: Effects of spatial fragmentation on the elevational distribution of bird diversity in a mountain adjacent to urban areas
Source: Ecol Evol. 2022 Jul 4;12(7):e9051. doi: 10.1002/ece3.9051 (PMC9251884; doi:10.1002/ece3.9051)
Supplement: Supplementary file 1 — TABLE S1Land use and land cover (LULC) types [file ECE3-12-e9051-s001.docx]

TABLE S1 Land use and land cover (LULC) types

| **LULC type** | **Abbreviation** | **Description** |
| --- | --- | --- |
| Forest | FOR | Forest area with tree canopy density ≥0.2 |
| Shrubland | SHR | Forest area with shrub coverage ≥40% |
| Woodland | WOO | Including open forest land (forest area with tree canopy density ≥0.1 and＜0.2), young afforested land, slash, nursery garden |
| Water | WAT | Continental water areas, ditches, hydraulic structures |
| Tourist area | TOU | Land for commerce and service industry |
| Construction land | CON | Housing estate, towns, including land for residence, industry, warehouse, governmental organ, school, park |
| Undeveloped land | UND | Other types of land, including all lands unused in towns, villages, industrial and mining sites |

TABLE S2 The detail of different line transects

|  |  | **Starting point** | | **End point** | |  |  |
| --- | --- | --- | --- | --- | --- | --- | --- |
| **No** | **Length/km** | **Longitude** | **Latitude** | **Longitude** | **Latitude** | **Elevation/m** | **Mountain belt** |
| L1 | 2.84 | 117.04778 | 36.27404 | 117.06522 | 36.27430 | 423-527 | Low, middle |
| L2 | 3.20 | 117.06541 | 36.27302 | 117.07489 | 36.25435 | 522-752 | Middle |
| L3 | 1.65 | 117.08179 | 36.30362 | 117.09197 | 36.30883 | 454-619 | Low, middle |
| L4 | 1.60 | 117.10201 | 36.25772 | 117.10827 | 36.26285 | 1216-1511 | High |
| L5 | 1.59 | 117.09835 | 36.25496 | 117.10470 | 36.25638 | 1408-1512 | High |
| L6 | 2.75 | 117.11196 | 36.24258 | 117.12810 | 36.24380 | 395-989 | Low, middle |
| L7 | 1.89 | 117.13069 | 36.24425 | 117.14064 | 36.24737 | 398-566 | Low, middle |
| L8 | 2.20 | 117.13558 | 36.21375 | 117.14078 | 36.22525 | 231-374 | Low |
| L9 | 2.10 | 117.05525 | 36.21618 | 117.05330 | 36.22742 | 222-326 | Low |
| L10 | 2.32 | 117.00761 | 36.22056 | 117.00614 | 36.21303 | 146-166 | Low |

TABLE S3 Bird list of Mount Tai

| **No** | **English name** | **Latin** | **Abbreviation** | **Number** | **IUCN Red List** |
| --- | --- | --- | --- | --- | --- |
| 1 | Chinese Spot-billed Duck | *Anas zonorhyncha* | ANZO | 26 | LC |
| 2 | Mallard | *Anas platyrhynchos* | ANPL | 4 | LC |
| 3 | Common Pheasant | *Phasianus colchicus* | PHCO | 75 | LC |
| 4 | Common Swift | *Apus apus* | APAP | 16 | LC |
| 5 | Pacific Swift | *Apus pacificus* | APPA | 2 | LC |
| 6 | Chestnut-winged Cuckoo | *Clamator coromandus* | CLCO | 2 | LC |
| 7 | Asian Koel | *Eudynamys scolopaceus* | EUSC | 59 | LC |
| 8 | Large Hawk-Cuckoo | *Hierococcyx sparverioides* | HISP | 10 | LC |
| 9 | Asian Lesser Cuckoo | *Cuculus poliocephalus* | CUPO | 1 | LC |
| 10 | Indian Cuckoo | *Cuculus micropterus* | CUMI | 36 | LC |
| 11 | Himalayan Cuckoo | *Cuculus saturatus* | CUSA | 4 | LC |
| 12 | Common Cuckoo | *Cuculus canorus* | CUCA | 50 | LC |
| 13 | Oriental Turtle Dove | *Streptopelia orientalis* | STOR | 98 | LC |
| 14 | Spotted Dove | *Spilopelia chinensis* | STCH | 57 | LC |
| 15 | Common Moorhen | *Gallinula chloropus* | GACH | 7 | LC |
| 16 | Eurasian Coot | *Fulica atra* | FUAT | 1 | LC |
| 17 | Little Grebe | *Tachybaptus ruficollis* | TARU | 7 | LC |
| 18 | Grey-headed Lapwing | *Vanellus cinereus* | VACI | 1 | LC |
| 19 | Little Ringed Plover | *Charadrius dubius* | CHDU | 1 | LC |
| 20 | Common Snipe | *Gallinago gallinago* | GAGA | 1 | LC |
| 21 | Common Sandpiper | *Actitis hypoleucos* | ACHY | 1 | LC |
| 22 | Green Sandpiper | *Tringa ochropus* | TROC | 1 | LC |
| 23 | Black-headed Gull | *Chroicocephalus ridibundus* | CHRI | 1 | LC |
| 24 | Great Cormorant | *Phalacrocorax carbo* | PHCA | 1 | LC |
| 25 | Yellow Bittern | *Ixobrychus sinensis* | LXSI | 1 | LC |
| 26 | Black-crowned Night Heron | *Nycticorax nycticorax* | NYNY | 14 | LC |
| 27 | Chinese Pond Heron | *Ardeola bacchus* | ARBA | 10 | LC |
| 28 | Grey Heron | *Ardea cinerea* | ARCI | 1 | LC |
| 29 | Little Egret | *Egretta garzetta* | EGGA | 11 | LC |
| 30 | Crested Honey-buzzard | *Pernis ptilorhynchus* | PEPT | 1 | LC |
| 31 | Black Baza | *Aviceda leuphotes* | AVLE | 3 | LC |
| 32 | Crested Goshawk | *Accipiter trivirgatus* | ACTR | 2 | LC |
| 33 | Chinese Sparrowhawk | *Accipiter soloensis* | ACSO | 19 | LC |
| 34 | Eurasian Sparrowhawk | *Accipiter nisus* | ACNI | 10 | LC |
| 35 | Pied Harrier | *Circus melanoleucos* | CIME | 1 | LC |
| 36 | Grey-faced Buzzard | *Butastur indicus* | BUIN | 5 | LC |
| 37 | Oriental Scops Owl | *Otus sunia* | OTSU | 8 | LC |
| 38 | Collared Scops Owl | *Otus lettia* | OTLE | 2 | LC |
| 39 | Common Hoopoe | *Upupa epops* | UPEP | 21 | LC |
| 40 | Oriental Dollarbird | *Eurystomus orientalis* | EUOR | 1 | LC |
| 41 | Black-capped Kingfisher | *Halcyon pileata* | HAPI | 11 | LC |
| 42 | Common Kingfisher | *Alcedo atthis* | ALAT | 9 | LC |
| 43 | Grey-capped Pygmy Woodpecker | *Yungipicus canicapillus* | DECA | 16 | LC |
| 44 | Great Spotted Woodpecker | *Dendrocopos major* | DEMA | 52 | LC |
| 45 | Grey-headed Woodpecker | *Picus canus* | PICA | 18 | LC |
| 46 | Common Kestrel | *Falco tinnunculus* | FATI | 11 | LC |
| 47 | Amur Falcon | *Falco amurensis* | FAAM | 1 | LC |
| 48 | Eurasian Hobby | *Falco subbuteo* | FASU | 2 | LC |
| 49 | Ashy Minivet | *Pericrocotus divaricatus* | PEDI | 1 | LC |
| 50 | Bull-headed Shrike | *Lanius bucephalus* | LABU | 1 | LC |
| 51 | Brown Shrike | *Lanius cristatus* | LACR | 12 | LC |
| 52 | Long-tailed Shrike | *Lanius schach* | LASC | 2 | LC |
| 53 | Black-naped Oriole | *Oriolus chinensis* | ORCH | 31 | LC |
| 54 | Hair-crested Drongo | *Dicrurus hottentottus* | DIHO | 24 | LC |
| 55 | Black Drongo | *Dicrurus macrocercus* | AIMA | 45 | LC |
| 56 | Azure-winged Magpie | *Cyanopica cyanus* | CYCY | 47 | LC |
| 57 | Red-billed Blue Magpie | *Urocissa erythroryncha* | URRI | 8 | LC |
| 58 | Eurasian Magpie | *Pica pica* | PIPI | 169 | LC |
| 59 | Red-billed Chough | *Pyrrhocorax pyrrhocorax* | PYPY | 5 | LC |
| 60 | Grey-headed Canary Flycatcher | *Culicicapa ceylonensis* | CUCE | 1 | LC |
| 61 | Marsh Tit | *Poecile palustris* | PAPA | 7 | LC |
| 62 | Cinereous Tit | *Parus cinereus* | PACN | 118 | NR |
| 63 | Chinese Penduline Tit | *Remiz consobrinus* | RECO | 1 | LC |
| 64 | Collared Finchbill | *Spizixos semitorques* | SPSE | 16 | LC |
| 65 | Light-vented Bulbul | *Pycnonotus sinensis* | PYSI | 111 | LC |
| 66 | Barn Swallow | *Hirundo rustica* | HIRU | 20 | LC |
| 67 | Red-rumped Swallow | *Cecropis daurica* | CEDA | 5 | LC |
| 68 | Rufous-faced Warbler | *Abroscopus albogularis* | ABAL | 1 | LC |
| 69 | Manchurian Bush Warbler | *Horornis canturians* | HOCA | 43 | LC |
| 70 | Brownish-flanked Bush Warbler | *Horornis fortipes* | HOFO | 4 | LC |
| 71 | Silver-throated Bushtit | *Aegithalos glaucogularis* | AEGL | 26 | LC |
| 72 | Black-throated Bushtit | *Aegithalos concinnus* | AECO | 2 | LC |
| 73 | Yellow-browed Warbler | *Phylloscopus inornatus* | PHIN | 13 | LC |
| 74 | Chinese Leaf Warbler | *Phylloscopus yunnanensis* | PHYU | 23 | LC |
| 75 | Pallas's Leaf Warbler | *Phylloscopus proregulus* | PHPR | 9 | LC |
| 76 | Yellow-streaked Warbler | *Phylloscopus armandii* | PHAR | 6 | LC |
| 77 | Claudia's Leaf Warbler | *Phylloscopus claudiae* | PHCL | 9 | LC |
| 78 | Oriental Reed Warbler | *Acrocephalus orientalis* | ACOR | 13 | LC |
| 79 | Black-browed Reed Warbler | *Acrocephalus bistrigiceps* | ACBI | 5 | LC |
| 80 | Zitting Cisticola | *Cisticola juncidis* | CIJU | 3 | LC |
| 81 | Beijing Hill Babbler | *Rhopophilus pekinensis* | RHPE | 1 | LC |
| 82 | Vinous-throated Parrotbill | *Sinosuthora webbiana* | SIWE | 39 | LC |
| 83 | Chestnut-flanked White-eye | *Zosterops erythropleurus* | ZOER | 3 | LC |
| 84 | Japanese White-eye | *Zosterops japonicus* | ZOJA | 45 | LC |
| 85 | Elliot's Laughingthrush | *Trochalopteron elliotii* | TREL | 9 | LC |
| 86 | Hwamei | *Garrulax canorus* | GACA | 51 | LC |
| 87 | Greater Necklaced Laughingthrush | *Pterorhinus pectoralis* | GAPE | 37 | LC |
| 88 | Eurasian Wren | *Troglodytes troglodytes* | TRTR | 48 | LC |
| 89 | White-cheeked Starling | *Spodiopsar cineraceus* | SPCI | 6 | LC |
| 90 | White's Thrush | *Zoothera aurea* | ZOAU | 2 | LC |
| 91 | Chinese Blackbird | *Turdus mandarinus* | TUMA | 17 | LC |
| 92 | Asian Brown Flycatcher | *Muscicapa dauurica* | MUDA | 5 | LC |
| 93 | Siberian Blue Robin | *Larvivora cyane* | LACY | 1 | LC |
| 94 | Rufous-tailed Robin | *Larvivora sibilans* | LASI | 1 | LC |
| 95 | White-bellied Redstart | *Luscinia phaenicuroides* | LUPH | 10 | LC |
| 96 | Blue Whistling Thrush | *Myophonus caeruleus* | MYCA | 8 | LC |
| 97 | Yellow-rumped Flycatcher | *Ficedula zanthopygia* | FIZA | 4 | LC |
| 98 | Daurian Redstart | *Phoenicurus auroreus* | PHAU | 104 | LC |
| 99 | Plumbeous Water Redstart | *Phoenicurus fuliginosus* | RHFU | 4 | LC |
| 100 | Blue Rock Thrush | *Monticola solitarius* | MOSO | 7 | LC |
| 101 | Russet Sparrow | *Passer cinnamomeus* | PACI | 90 | LC |
| 102 | Eurasian Tree Sparrow | *Passer montanus* | PAMO | 96 | LC |
| 103 | Siberian Accentor | *Prunella montanella* | PRMO | 1 | LC |
| 104 | Forest Wagtail | *Dendronanthus indicus* | DEIN | 3 | LC |
| 105 | Grey Wagtail | *Motacilla cinerea* | MOCI | 35 | LC |
| 106 | White Wagtail | *Motacilla alba* | MOAL | 17 | LC |
| 107 | Chinese Grosbeak | *Eophona migratoria* | EOMI | 2 | LC |
| 108 | Grey-capped Greenfinch | *Chloris sinica* | CHSI | 40 | LC |
| 109 | Meadow Bunting | *Emberiza cioides* | EMCI | 49 | LC |
| 110 | Tristram's Bunting | *Emberiza tristrami* | EMTR | 1 | LC |
| 111 | Little Bunting | *Emberiza pusilla* | EMPS | 1 | LC |
| 112 | Yellow-browed Bunting | *Emberiza chrysophrys* | EMCH | 1 | LC |
| 113 | Yellow-throated Bunting | *Emberiza elegans* | EMEL | 5 | LC |

TABLE S4 The bird community at different altitudes

| **Habitat types** | **Abbreviation** | **Species** | **Individuals** | **Shannon-Wiener index** |
| --- | --- | --- | --- | --- |
| Forest | For | 72 | 3117 | 3.29 |
| Tourist area | Tou | 2 | 113 | 0.12 |
| Water | Wat | 32 | 406 | 2.73 |
| Undeveloped land | Und | 5 | 24 | 1.53 |
| Shrub | Shr | 48 | 1485 | 2.99 |
| Woodland | Woo | 23 | 500 | 1.93 |
| Construction land | Con | 9 | 66 | 1.6 |
